# Site-Directed Mutagenesis from Arg195 to His of a Microalgal Putatively Chloroplastidial Glycerol-3-Phosphate Acyltransferase Causes an Increase in Phospholipid Levels in Yeast

**DOI:** 10.3389/fpls.2016.00286

**Published:** 2016-03-10

**Authors:** Long-Ling Ouyang, Hui Li, Xiao-Jun Yan, Ji-Lin Xu, Zhi-Gang Zhou

**Affiliations:** ^1^College of Aqua-Life Science and Technology, Shanghai Ocean UniversityShanghai, China; ^2^Department of Biology and Food Engineering, Bengbu UniversityBengbu, China; ^3^Key Laboratory of Applied Marine Biotechnology, Ningbo UniversityNingbo, China

**Keywords:** *Lobosphaera incisa* H4301, glycerol-3-phosphate acyltransferase (GPAT), plastid, site-directed mutagenesis, UPLC-Q-TOF-MS, glycerolipid

## Abstract

To analyze the contribution of glycerol-3-phosphate acyltransferase (GPAT) to the first acylation of glycerol-3-phosphate (G-3-P), the present study focused on a functional analysis of the GPAT gene from *Lobosphaera incisa* (designated as *LiGPAT*). A full-length cDNA of *LiGPAT* consisting of a 1,305-bp ORF, a 1,652-bp 5′-UTR, and a 354-bp 3′-UTR, was cloned. The ORF encoded a 434-amino acid peptide, of which 63 residues at the N-terminus defined a chloroplast transit peptide. Multiple sequence alignment and phylogeny analysis of GPAT homologs provided the convincible bioinformatics evidence that LiGPAT was localized to chloroplasts. Considering the conservation of His among the G-3-P binding sites from chloroplastidial GPATs and the substitution of His by Arg at position 195 in the LiGPAT mature protein (designated mLiGPAT), we established the heterologous expression of either *mLiGPAT* or its mutant (Arg195His) (*sdmLiGPAT*) in the GPAT-deficient yeast mutant *gat1*Δ. Lipid profile analyses of these transgenic yeasts not only validated the acylation function of *LiGPAT* but also indicated that the site-directed mutagenesis from Arg^195^ to His led to an increase in the phospholipid level in yeast. Semi-quantitative analysis of mLiGPAT and sdmLiGPAT, together with the structural superimposition of their G-3-P binding sites, indicated that the increased enzymatic activity was caused by the enlarged accessible surface of the phosphate group binding pocket when Arg^195^ was mutated to His. Thus, the potential of genetic manipulation of GPAT to increase the glycerolipid level in *L*. *incisa* and other microalgae would be of great interest.

## Introduction

In plants, *de novo* biosynthesis of fatty acids occurs exclusively in chloroplasts. The fatty acids generated are either directly metabolized into glycolipids and PG within the chloroplast or exported across the envelope to the ER to form phospholipids and neutral lipids. To synthesize these glycerolipids in both chloroplast and ER, glycerol-3-phosphate acyltransferase (GPAT, E.C. 2.3.1.15) is first required to acylate fatty acids in the glycerol backbone of G-3-P. This enzyme localized in ER was demonstrated to be crucial for cutin, suberin, or storage oil biosynthesis in *Arabidopsis* *thaliana* (Zheng et al., 2003; Gidda et al., 2009), *Ricinus* *communis* (Cagliari et al., 2010) and *Brassica* *napus* (Chen et al., 2010). In addition, it was found that a deficiency in the chloroplastidial GPAT activity could cause a reduction (10–25%) in the PG content of *Arabidopsis* (Kunst et al., 1988; Xu et al., 2006). Thus, GPAT has been found to play a pivotal role in initiating all glycerolipid biosynthesis in higher plants. In comparison, functional analyses of GPAT from microalgae are rare.

To understand the features of the first step of glycerolipid biosynthesis catalyzed by GPAT in microalgae, we attempted to identify one cloned GPAT gene from an oleaginous green microalga, *Lobosphaera incisa* Reisigl (designated as LiGPAT). This microalga possesses a high content of photosynthetic membrane lipids as suggested by a large incised chloroplast with many parallel thylakoid membranes (Merzlyak et al., 2007; Ouyang et al., 2012, 2013b), and it has the ability to accumulate TAG to form oil bodies in cells, especially under nitrogen starvation (Khozin-Goldberg et al., 2002; Zhang et al., 2002; Tong et al., 2011; Ouyang et al., 2013b). Thus, the study of the function of the GPAT gene from *L*. *incisa* might indicate the role of GPAT in microalgae. Given that GPAT in plants can localize to the chloroplast or the ER, the subcellular localization of the encoded protein LiGPAT was analyzed by bioinformatics technique. Heterologous complementation in a GPAT deficient mutant of yeast, *gat1*Δ (Zheng and Zou, 2001), was used to validate the function of *LiGPAT*, and the yeast lipids were analyzed by lipidomic approaches using UPLC-ESI-Q-TOF-MS and multivariate data analysis. Surprisingly, we found that the conserved His in the G-3-P binding sites from chloroplastidial GPATs was substituted by Arg at position 195 in this chloroplastidial LiGPAT mature protein, and site-directed mutagenesis at this site of LiGPAT improved the phospholipid level in yeast. These findings help us to understand the characteristics of a putatively chloroplastidial GPAT in *L*. *incisa* and thus provide a strategy for genetic engineering to improve the microalgae-based production of biofuels.

## Materials and Methods

### Strains, Medium and Growth Conditions

*Lobosphaera incisa*, deposited in the Culture Collection of Algae of Charles University in Prague under ID H4301 was cultivated in BG-11 medium (Stanier et al., 1971) in 500-mL glass flasks as described previously (Ouyang et al., 2013b). During culture, the flasks were shaken several times a day by hand at regular intervals.

### Cloning of cDNA and DNA Encoding *LiGPAT*

A pair of degenerate primers (G1 and G2) (**Supplementary Table S1**) for the LiGPAT gene cDNA cloning were designed based on the amino acid sequences of GPAT from *Ostreococcus tauri* (GenBank Accession Number 116061306) and *Chlamydomonas reinhardtii* (GenBank Accession Number 159473711). Total RNA isolated by TRIzol reagent (Invitrogen) from *L*. *incisa* was used to synthesize cDNA with a Reverse Transcribed Kit II (TaKaRa). The full-length cDNA of *LiGPAT* was amplified by a SMART^TM^ RACE cDNA Amplification Kit (Clontech). Two gene-specific primers (NGSP5-1 and GSP5-2) for the first 5′-RACE reaction, one gene-specific primer (GSP5-4) for the second 5′-RACE reaction, and two gene-specific primers (NGSP3-1 and GSP3-2) for the 3′-RACE reaction were designed (**Supplementary Table S1**). Genomic DNA extracted by the CTAB method from *L*. *incisa* (Dellaporta et al., 1983) was used to amplify both the coding region and the untranslated region of *LiGPAT* with four pairs of primers (**Supplementary Table S1**). All PCR products of the expected size were cloned into the pMD19-T cloning vector (TaKaRa). The resulting constructs were transformed into *Escherichia coli* DH5α and verified by sequencing. The BLAST Server^2^ was used to annotate the cloned sequences.

### Southern Blot Analysis of *LiGPAT*

Genomic DNA of *L*. *incisa* was double digested with *Xho*I/*Not*I or *Hind*III/*Not*I restriction endonucleases at 37°C for 4-6 h. The digested DNA samples were fractionated on a 1.0% agarose gel and then transferred to a NC filter membrane (Millipore). A pair of primers was designed based on the conserved domain of GPAT (**Supplementary Table S1**). A 311-bp biotin-labeled DNA sequence was prepared to use as a probe with a North2South^®^ Biotin Random Prime Labeling Kit (Thermo Scientific). Subsequently, the hybridization was detected by the standard Southern blot procedure (Sambrook and Russell, 2001) with a North2South Chemiluminescent Hybridization and Detection Kit (Thermo Scientific). Signals were visualized by exposure to XBT-1 film (Kodak) at room temperature for 60-120 s.

### Bioinformatics Analysis

The intron and exon regions from *LiGPAT* were analyzed using Spidey^3^. Signal peptide sites of the amino acid sequence of LiGPAT were predicted by the SignalP 4.1 Server^4^, and the transit peptide sites were predicted by the TargetP 1.1 Server^5^ and the ChloroP 1.1 Server^6^. Conserved domains were searched in NCBI’s CDD (Marchler-Bauer et al., 2011). The PredictProtein program^7^ was applied to predict protein structural and functional features (Rost et al., 2004). Protein structures were performed with I-TASSER^8^ (Roy et al., 2010). The superimposed images of the LiGPAT tertiary structure were obtained from SuperPose 1.0^9^ (Maiti et al., 2004) and displayed with UCSF Chimera 1.10 (Pettersen et al., 2004).

### Multiple Sequence Alignment of GPAT Homologs

The available chloroplastidial GPAT amino acid sequences of *Arabidopsis thaliana* (GenBank Accession Number Q43307), *Auxenochlorella protothecoides* (GenBank Accession Number KFM22407), *Chlamydomonas reinhardtii* (GenBank Accession Number XP_001694977), *Coccomyxa subellipsoidea* C-169 (GenBank Accession Number XP_005643353), *Cucurbita moschata* (GenBank Accession Number BAB17755), *Cyanidioschyzon merolae* Strain 10D (GenBank Accession Number XP_006587606), *Glycine max* (GenBank Accession Number XP_006587606), *Micromonas pusilla* CCMP 1545 (GenBank Accession Number XP_003060587), *Micromonas* sp. RCC299 (GenBank Accession Number XP_002505030), *Ostreococcus lucimarinus* (GenBank Accession Number ABO94442), *Ostreococcus tauri* (GenBank Accession Number CAL52024), *Phaeodactylum tricornutum* (GenBank Accession Number XP_002184838), *Ricinus communis* (GenBank Accession Number XP_002518993), *Thalassiosira pseudonana* (GenBank Accession Number XP_002292905), and *Volvox carteri* f. *nagariensis* (GenBank Accession Number XP_002950506) were retrieved from GenBank. The amino acid sequences of the ER-bound GPAT isoform 4 (GenBank accession number Q9LMM0), isoform 5 (GenBank Accession Number NP_187750), isoform 6 (GenBank Accession Number NP_181346), and isoform 8 (GenBank Accession Number NP_191950) from *Arabidopsis thaliana*, and ER-bound GPAT from *Medicago truncatula* (GenBank Accession Number AES79440) and *Ricinus communis* (GenBank Accession Number XP_002511873) were also obtained from GenBank. Multiple sequence alignment of the chloroplastidial GPATs and the ER-bound GPATs were performed with the ClustalX program (Thompson et al., 1997). The web-based BLAST2 program (Altschul et al., 1990) at NCBI was employed to generate pairwise similarity scores of the aligned sequences. The Multiple EM for Motif Elicitation (MEME) program (Bailey et al., 2006) was used to identify conserved sequence motifs.

### Phylogeny Inference

The amino acid sequences of GPAT as well as LPAAT from higher plants and microalgae were retrieved from GenBank. Three GPATs from *Nitrosococcus halophilus*, *Bradyrhizobium japonicum*, and *Ralstonia pickettii* DTP0602 were chosen as an arbitrary outgroup. All accession numbers are presented in the phylogeny tree. All of the conserved domain sequences annotated by searching CDD were also aligned with the ClustalX program (Thompson et al., 1997). Phylogenetic analysis was conducted using maximum likelihood (ML) methods with MEGA 6.0 (Tamura et al., 2013) by using the most appropriate model (LG + G + Γ) determined by ProtTest v3.3 (Darriba et al., 2011). Branch points were tested for significance by bootstrapping with 1,000 replications (Felsenstein, 1985; Tamura et al., 2013).

### Construction of *mLiGPAT* and *sdmLiGPAT* Expression Plasmids

To construct a bacterial plasmid, the 1,131-bp of the LiGPAT mature protein coding gene (designated as *mLiGPAT*) with *Bam*HI/*Xho*I digestion sites was amplified with a pair of primers (EcBamF and XhoR) (**Supplementary Table S1**). The PCR products were sticky-ended and subcloned into the *Bam*HI/*Xho*I sites of pET 28a to obtain the plasmid pET-mLiG.

For heterologous expression in yeast, *mLiGPAT* and the site-directed mutation (Arg195His) of *mLiGPAT* (designated as *sdmLiGPAT*) expression plasmids were constructed. An approximately 1.2 kb cDNA fragment containing the *Bam*HI/*Xho*I digestion sites of *mLiGPAT* was amplified with the primers ScBamF and XhoR (**Supplementary Table S1**). Site-directed mutagenesis by splicing overlap extension PCR (SOE-PCR) (Ge and Rudolph, 1997) was performed to generate *sdmLiGPAT* with *Bam*HI/*Xho*I digestion sites using four primers ScBamF, MuScR, XhoR, and MuScF (**Supplementary Table S1**). The PCR products of *mLiGPAT* and *sdmLiGPAT* were sticky-ended and subcloned into the *Bam*HI/*Xho*I sites of pYES2 (Invitrogen) to obtain the plasmids pY-mLiG and pY-sdmLiG, respectively. Prior to transforming the resulting plasmids into host cells, the correct orientation and in-frame fusion of all of the inserts was verified by sequencing.

### Heterologous Expression of *LiGPAT*

To obtain soluble, recombinant mLiGPAT protein, the plasmid pET-mLiG and the vector pET 28a as a control were introduced into *E*. *coli* BL21 (DE3) pLysS (designated pmLiG/BL and pET/BL), respectively. Single colonies of pmLiG/BL or pET/BL were inoculated in LB medium. After incubation at 37°C for 12 h, the cells were collected and resuspended in LB medium with 1 mM IPTG to obtain an OD600 of 0.6. After incubation at 18°C overnight (Schein and Noteborn, 1988), soluble recombinant protein with a His-tag was expressed and detected by SDS-PAGE.

To express *mLiGPAT* and *sdmLiGPAT* in yeast for functional identification, we chose the GPAT deficient yeast mutant, *gat1*Δ (*BY4742*, *Matα*, *his3Δ1*, *leu2Δ0*, *lys2Δ0*, *ura3Δ0*, *YKR067w:*:*kanMX4*) as described by Zheng and Zou (2001). This strain was purchased from EUROSCARF^10^. Single colonies carrying pYES2 (plasmid-only as a control) or pY-mLiG or pY-sdmLiG (designated gPY, gmLiGPAT, and gsdmLiGPAT, respectively) were inoculated into SC-uracil medium with 2% glucose. The parental strain of *gat1*Δ, BY4742 (*Matα*, *his3Δ1*, *leu2Δ0*, *lys2Δ0*, *ura3Δ0*), was used as a positive control, while *gat1*Δ was used as a negative control. After incubation at 30°C for 30 h, cells of *gat1*Δ, gPY, BY4742, gmLiGPAT, and gsdmLiGPAT were collected by centrifugation and resuspended in SC-uracil medium with 2% galactose. After incubation at 30°C for 12 h to reach to the logarithmic phase, the yeast cells were transferred to 16°C and incubated for another 48 h. Cells were harvested by centrifugation at 4°C. For preparation of the yeast homogenates, the cell pellets were washed with 10 volumes of distilled H_2_O and then immediately frozen in liquid nitrogen and stored at -80°C until use. For total lipid extraction, the cell pellets were lyophilized and stored at -20°C until use. Each sample was collected in duplicate. Colony PCR of each sample was accomplished using the primers pYF and pYR (**Supplementary Table S1**) to ensure the insertion of the target gene.

### Polyclonal Antibody Preparation and Purification

Soluble recombinant mLiGPAT protein was purified by Ni-affinity column chromatography (Bio-Rad) and verified by HPLC-MS. New Zealand rabbits were immunized for LiGPAT polyclonal antibody preparation. The purified mLiGPAT protein was electrophoretically transferred from SDS polyacrylamide gels to NC membranes (Millipore) for polyclonal antibody preparation (Smith and Fisher, 1984). Next, the NC blots were incubated for l h in 3% BSA in PBS before an additional incubation in the prepared LiGPAT polyclonal antibody for 16 h at 4°C. Afterward, the blots were washed several times with PBS and eluted with 0.2 M glycine-HCl (pH 2.3) for 20 min. The eluate was immediately neutralized by the addition of 1 M Tris-HCl and PBS and stored at -20°C until use.

### Western Blot Analysis

Fresh cells of *L*. *incisa* were ground in liquid nitrogen, resuspended in 50-100 μL breaking buffer (25 mM Tris-HCl, pH 6.5, 50 mM NaCl, 2 mM β-mercaptoethanol) and vortexed. Frozen cell pellets of yeast were resuspended in 500 μL breaking buffer (50 mM PBS, pH 7.4, 1 mM EDTA, 5% glycerol, 1 mM PMSF) and lysed by shear force using acid-washed glass beads according to the user manual (Invitrogen). Frozen cell pellets of *E*. *coli* were resuspended in 0.1 M PBS buffer and then sonicated on ice with a probe sonicator until the suspension was partially clear.

The lysed cells were centrifuged at 20,000 × *g* at 4°C, and the supernatant was collected and stored at -80°C until use. The protein concentration was determined by the Bradford protein assay (Bradford, 1976).

Crude proteins from *L*. *incisa*, *E*. *coli* or yeast were electrophoretically transferred to NC membranes (Millipore) as described above, and the Western blots were performed according to the standard protocol (Sambrook and Russell, 2001). The purified LiGPAT polyclonal antibody and the secondary antibody, peroxidase-conjugated goat anti-rabbit IgG (Shanghai Youke Biotechnology Co., Ltd.), were appropriately diluted. Immunoreactive bands were visualized by the addition of DAB according to the manufacturer’s manual (Tiangen). The levels of mLiGPAT and sdmLiGPAT expressed in yeast were semi-quantified by measuring the band intensity on their corresponding blots with ImageJ software^11^.

### Total Lipid Extraction and Fractionation

Total lipid from yeast was extracted according to Bligh and Dyer (1959) with minor modifications. Acid washed glass beads with a diameter of 0.4-0.6 mm (Omega Bio-Tek) were used to break the cell walls. The extent of lysis was observed with a microscope, keeping the degree of breakage of each sample the same as far as possible.

Phospholipids from the extracted total lipids of approximately 50 mg lyophilized yeast were separated using solid-phase extraction (Christie and Han, 2010). A 500 mg cartridge of silica gel (CNW) was first conditioned by elution with 5 mL of chloroform, and the total lipids from yeast were then applied to it. Elution with 10 mL of methanol yielded the phospholipids. This fraction was concentrated under a stream of nitrogen gas and then weighed.

### UPLC-ESI-Q-TOF-MS Analysis

Reversed-phase analysis of lipids was performed on a Waters ACQUITY UPLC system using an ACQUITY UPLC BEH C8 analytical column (i.d. 2.1 × 100 mm, particle size 1.7 μm). The temperature of the sample chamber was set at 4°C, the column temperature was set at 40°C, and the injection volume was 4 μL for each analysis. A 1:4 split of the column effluent was used to achieve a flow rate of approximately 0.35 mL/min into the ESI source. To produce ions that could be readily fragmented, 0.001% lithium acetate and 0.1% formic acid were added to the mobile phase as the electrolyte. For efficient separation of the total lipids, water/tetrahydrofuran (3:1, v/v) was used as the mobile phase A and acetonitrile/methanol/tetrahydrofuran (2:1:1, v/v/v) as the mobile phase B. The initial composition of the mobile phase B was changed from 40 to 70% in 10 min and held for 7 min, then increased to 100% in 6 min and held for 1 min, and finally returned to the initial 40% in 1 min and equilibrated for 10 min. MS analysis was performed in a negative ion mode on a Waters Q-TOF Premier mass spectrometer. The mass range was from 100 to 1,200 with a scan duration of 0.3 s and an interscan delay of 0.02 s. High-purity nitrogen was used as the nebulizer and drying gas at a constant flow rate of 50 L/h, and the source temperature was set at 120°C. The capillary voltage was set at 2.6 kV, and the sampling cone voltage was set at the ramp of 35–80 V. MS/MS analysis was performed at a collision energy range of 25-35 V with argon as the collision gas. The TOF analyzer was used in a V mode and tuned for maximum resolution (>10,000 resolving power at m/z 1,000). Prior to the experiment, the instrument was calibrated with sodium formate, and the lock mass spray for precise mass determination was set with leucine enkephalin at a concentration 400 ng/μL, generating an [M-H]^-^ ion at 554.2615 Da in ESI^-^ mode. The lock spray frequency was set at 10 s.

### Lipidomics Data Processing

The original data from the ESI^-^ mode were acquired by the UPLC-Q-TOF-MS system and analyzed by a MassLynx 4.1 data processing system (Waters). The MarkerLynx matrices with peak numbers [based on the RT and mass-to-charge ratio (m/z)], sample names, and normalized peak intensities were exported to SIMCA-P+ 12.0 (Umetrics) and analyzed by PCA, PLS-DA, and OPLS-DA. The quality of the models PLS-DA and OPLS-DA was evaluated by two parameters, R^2^Y(cum) and Q^2^(cum). R^2^Y(cum) is the cumulative fraction of the sum of squares of all Y-variables that the model can explain using the latent variables, indicating the explanative ability of the model. Q^2^(cum) depicts the cumulative fraction of the total variation that can be predicted using the model via sevenfold cross-validation, indicating the predictability of the model. In general, R^2^Y(cum) and Q^2^(cum) values close to 1.0 indicate an excellent fit to the model, and the difference between these two values should be less than 0.3 (Wiklund, 2008). If the value of the Q^2^(cum) is higher than 0.9, the model is considered an excellent one (Wiklund, 2008). CV-ANOVA was systematically performed based on the PLS-DA model to rule out the non-randomness of the separation between groups. Generally, in permutation tests with 999 iterations, the intercept value of *Q*^2^ > 0.05 indicates over-fit in the original model (Kang et al., 2008; Lu et al., 2012).

### Identification of Lipid Metabolites

Variables meeting two criteria, specifically, high VIP and CIJF_JK_ excluding zero, were selected as potential lipid biomarkers, which contributed to the separation between groups (Eriksson et al., 2006; Cai et al., 2015). The lipid metabolites were identified by the RT, m/z, and the characteristic fragment ions deduced by MS/MS (Yan et al., 2010). In addition, some public databases including HMDB^12^, LIPID MAPS^13^, and METLIN^14^ were also used to help elucidate the putative ion structures.

## Results

### Cloning and Characterization of the LiGPAT Gene

Based on the amino acid sequences of GPAT proteins available from *Ostreococcus tauri* and *Chlamydononas reinhardtii*, a pair of degenerate primers (**Supplementary Table S1**) was designed, with which a 321-bp cDNA fragment was amplified from *L*. *incisa*. BLAST analysis revealed that this sequence was a GPAT homolog, and so it was designated *LiGPAT*. Subsequently, a 3,278-bp full-length cDNA of this gene, consisting of a 1,305-bp ORF, a 1,619-bp 5′-UTR, and a 354-bp 3′-UTR was obtained by the RACE technique. The nucleotide sequence of *LiGPAT* was identical to the unique annotated GPAT gene from the transcriptome of *L*. *incisa* (Ouyang et al., 2013a). A comparison of the cDNA sequence with its corresponding DNA sequence (**Supplementary Figure S1A**) revealed that the gene contained seven introns. The introns, 279, 371, 404, 145, 307, 211, and 745 bp beginning from the 5′-end, contained splice sites that all conformed to the GT-AG rule. Both the cDNA and the DNA sequences of *LiGPAT* were deposited in GenBank under the accession numbers KM670441 and KM670442, respectively. Southern blot analysis of the genomic DNA digested by either *Not*I/*Xho*I or *Not*I/*Hind*III using a 311-bp specific probe suggested that *LiGPAT* was a single copy gene in *L*. *incisa* (**Supplementary Figure S1B**). The same result for nucleus-encoded chloroplastidial GPAT genes was observed in a number of angiosperm families (Ishizaki et al., 1988; Kunst et al., 1988; Weber et al., 1991; Nishida et al., 1993; Bhella and Mackenzie, 1994).

**FIGURE 1:**
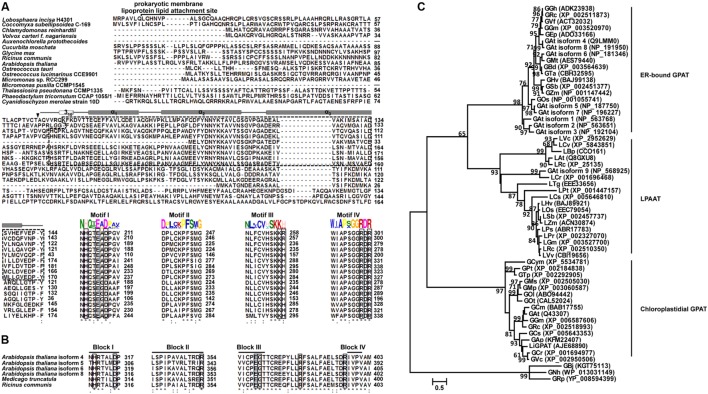
**Multiple sequence alignment and phylogeny analysis of GPAT homologs.** Identical amino acids are indicated by “^∗^”, and the important catalytic sites are boxed. **(A)** ClustalW alignment of the N-terminal sequences and four acyltransferase domain sequences from chloroplastidial GPATs. The identified GPAT_N domain is indicated by a dashed line box. The secondary structure assigned for the GPAT_N domain is represented by a cylinder (α-helix). A filled down arrow (▼) indicates the cleavage site of LiGPAT. The putative prokaryotic membrane lipoprotein lipid attachment site is indicated above the aligned sequences. Motifs I-IV were predicted by the MEME program. **(B)** ClustalW alignment of four acyltransferase domain sequences from ER-bound GPATs. Blocks I-IV were suggested by Lewin et al. (1999). **(C)** Phylogeny inference based on GPAT and LPAAT amino acid sequences. The phylogeny tree was inferred by the Maximum Likelihood (ML) method based on LG + G + Γ. The tree with the highest log likelihood (-6,958.7325) is shown. Bootstrap analysis was based on 1,000 re-samplings, and only support values higher than 60% are shown in the phylogeny. All accession numbers are presented in the phylogeny tree.

The ORF of *LiGPAT* encoded a 434-amino acid peptide, in which the domains GPAT_N (GenBank Accession Number cl20739) and LPLAT_GPAT (GenBank Accession Number cd07985) were annotated by searching CDD. A structural motif similar to a prokaryotic membrane lipoprotein lipid attachment site (Leu^8^- Cys^18^) was identified by the PredictProtein program (**Figure 1A**). This motif was also identified in a chloroplastidial form of the acetyl-CoA carboxylase of pea (Shorrosh et al., 1996) and a chloroplastidial NEF1 of *Arabidopsis thaliana* (Ariizumi et al., 2004). Neither the transmembrane domain nor the signal peptide in LiGPAT was predicted, whereas the N-terminal sequence of 63 residues was identified as a cTP (**Figure 1A**) as predicted by both the TargetP 1.1 Server and the ChloroP 1.1 Server. Characteristics of this cTP, including a high content (14.28%) of hydroxylated residues (Ser and Thr), a high content (19.05%) of hydrophobic residues (Ala and Val), the absence of the acidic residues (Asp and Glu), and very few Pro and Gly among the first 10 residues, were consistent with previously described cTP sequences (von Heijne et al., 1989). These results suggested that the LiGPAT might be a chloroplastidial GPAT.

### Alignment and Phylogeny Analysis of GPAT Homologs

To ascertain the features of chloroplastidial GPAT amino acid sequences, a pairwise sequence alignment and a complete multiple sequence alignment were carried out separately. The results of the pairwise alignment showed a higher similarity among chloroplastidial GPAT proteins from higher plant species (67-74%) than from microalgal species (33-79%). LiGPAT was more conserved with GPATs from other Trebouxiophyceae species (*Coccomyxa subellipsoidea* and *Auxenochlorella protothecoides*) and Chlorophyceae species (*Chlamydomonas reinhardtii* and *Volvox carteri*) (56-60%) than with Mamiellophyceae species (*Ostreococcus lucimarinus*, *Ostreococcus tauri*, *Micromonas pusilla*, and *Micromonas* sp.) (42-43%), Stramenopiles species (*Thalassiosira pseudonana* and *Phaeodactylum tricornutum*) (36-43%), and Rhodophyta species (*Cyanidioschyzon merolae*) (33%).

The complete multiple sequence alignment identified 39 fully conserved residues that corresponded to only 8.8% of the average 430 residues. The GPAT_N domain identified in LiGPAT was also found in GPATs from *Coccomyxa subellipsoidea*, *Chlamydomonas reinhardtii*, *Volvox carteri*, *Auxenochlorella protothecoides*, *Cucurbita moschata*, *Glycine max*, *Ricinus communis*, and *Arabidopsis thaliana* (**Figure 1A**). The length of the domain GPAT_N was similar (74-78 amino acid residues) except for that from *Auxenochlorella protothecoides*, due to the incomplete sequence, but its sequence was different from others (**Figure 1A**). In contrast, four motifs predicted by using the MEME program were relatively conserved (**Figure 1A**). The H(X)_4_D motif in Motif I was a conserved consensus sequence among many glycerolipid acyltransferases. The residues Lys, His, Arg, and Arg in Motif III and IV in chloroplastidial GPATs (**Figure 1A**) were considered to form a positive pocket to bind the phosphate group of G-3-P (Lewin et al., 1999; Turnbull et al., 2001). It is worth noting that these four G-3-P binding sites were well conserved except for the His residue, which was replaced by Arg in *L*. *incisa* and *Coccomyxa subellipsoidea* (**Figure 1A**).

The ER-bound GPATs were aligned, and four acyltransferase domains were identified (**Figure 1B**). Interestingly, these acyltransferase domains were significantly different from those of chloroplastidial GPATs, except the His and Asp residues from the H(X)_4_D motif in Block I (**Figure 1B**). The Gly residue in Block III and the Pro residue in Block IV, both of which were suggested to be catalytically important sites (Lewin et al., 1999), were completely conserved (**Figure 1B**). In addition, the residues Arg in Block II and Glu and Ser in Block III were invariant among the cytoplasmic GPATs for G-3-P binding (**Figure 1B**).

Although the previously defined conserved domains of GPAT were similar to those of LPAAT (Heath and Rock, 1998; Lewin et al., 1999; Slabas et al., 2002), the phylogenetic tree showed an apparently different phylogenetic support between lineages of GPAT and LPAAT except that the GPAT isoform 9 from *Arabidopsis thaliana* was in the LPAAT clade (**Figure 1C**). The ER-bound GPATs and the mitochondrial GPATs formed a cluster apart from the chloroplastidial one comprising both higher plants and microalgae (**Figure 1C**), and this result was in agreement with a previous report (Cagliari et al., 2010). This separation could be explained by the differences in the G-3-P binding and catalytically important sites between the cytoplasmic and the chloroplastidial GPATs as mentioned above. The phylogeny also suggested a sister group relationship between the subclade, consisting of diatoms and red algae, and the one comprising higher plants and green microalgae (**Figure 1C**), which was consistent with the sequence similarity among these species.

These multiple-sequence alignments and phylogeny indicated that LiGPAT possessed the sequence features that conformed to those of chloroplastidial GPATs, providing further evidence that LiGPAT was localized in *L*. *incisa* chloroplasts.

### Functional Identification of *LiGPAT* in a *gat1*Δ Mutant of Yeast

Multiple sequence alignment (**Figure 1A**) showed that the Arg^195^ in mLiGPAT was different from His, which was considered one of the G-3-P binding sites in most chloroplastidial GPATs. When this residue His was mutated to Ser, the biological activity of squash chloroplastidial GPAT decreased (Slabas et al., 2002). Accordingly, it was inferred that the catalytic ability of this LiGPAT might be different from (probably lower than) the one with His at position 195. Thus, to identify the function of *LiGPAT*, heterologous expression of *mLiGPAT* as well as its mutant (Arg195His) *sdmLiGPAT* generated by site-directed mutagenesis was performed in the GPAT-deficient yeast strain *gat1*Δ.

To identify the function of GPAT in yeast, the activity of this enzyme was determined *in vitro*, for example, by routinely using ^14^C-labeled G-3-P as described by Zheng and Zou (2001). Because of the inconvenience in other ordinary laboratories without any protection from irradiation, a metabolomics approach by using UPLC-ESI-Q-TOF-MS and multivariate data analysis (Fiehn et al., 2000; Wiklund et al., 2005; Van Assche et al., 2015) was employed in this study.

**FIGURE 2:**
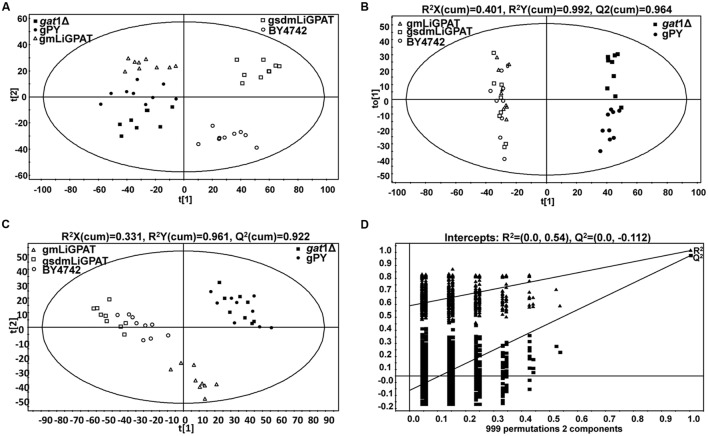
**Multivariate data analysis of lipidomics data from transgenic (gPY, gmLiGPAT, and gsdmLiGPAT), GPAT-deficient (*gat1*Δ), and parental (BY4742) yeasts in negative ion scan mode.** **(A)** PCA score plot of data from *gat1*Δ, gPY, gmLiGPAT, gsdmLiGPAT, and BY4742 for the first two components. **(B)** OPLS-DA score plot of data from the wild-type group (gmLiGPAT, gsdmLiGPAT, and BY4742) versus the deficient group (gPY and *gat1*Δ). **(C)** PLS-DA score plot of data from the wild-type group versus the deficient group. **(D)** Validation plot of PLS-DA analysis with the number of permutations equaling 999. *R*^2^ (filled triangle) is the explained variance, and *Q*^2^ (filled square) is an estimate of the predictive ability of the 7 models.

A PCA model with two-components was constructed, which showed that gsdmLiGPAT (site-directed mutated) clustered with the parental yeast strain BY4742 (this group was designated as the wild-type) but was clearly separated from the *gat1*Δ (GPAT-deficient) and plasmid-only control yeast gPY (this group was designated as deficient) (**Figure 2A**). In this PCA model, which could not be well validated, gmLiGPAT (mLiGPAT-transformed) did not significantly separate from the deficient group. In comparison, both the OPLS-DA [*R^2^Y(cum)* = 0.992 and *Q^2^(cum)* = 0.964] and the PLS-DA [*R^2^Y(cum)* = 0.961 and *Q^2^(cum)* = 0.922] models with high *R^2^Y(cum)* and *Q^2^(cum)* values could provide reliable support for the separation of gmLiGPAT and the wild-type group from the deficient group (**Figures 2B,C**). Validation of the PLS-DA model with the number of permutations equaling 999 generated intercepts of *R*^2^ = 0.54 and *Q*^2^ = -0.112 (**Figure 2D**), giving an additional proof of the statistically valid and well fit model because the intercept of Q^2^-point regression line was below zero. These statistical analyses indicated that lipid compositions of gmLiGPAT and gsdmLiGPAT indeed significantly differed from those of *gat1*Δ and gPY but were similar to those of the parental strain BY4742. It was concluded that the deficiency of GPAT in the mutant *gat1*Δ was corrected by the introduction of *LiGPAT*, thus confirming the acylation function of the GPAT protein from *L*. *incisa*.

**FIGURE 3:**
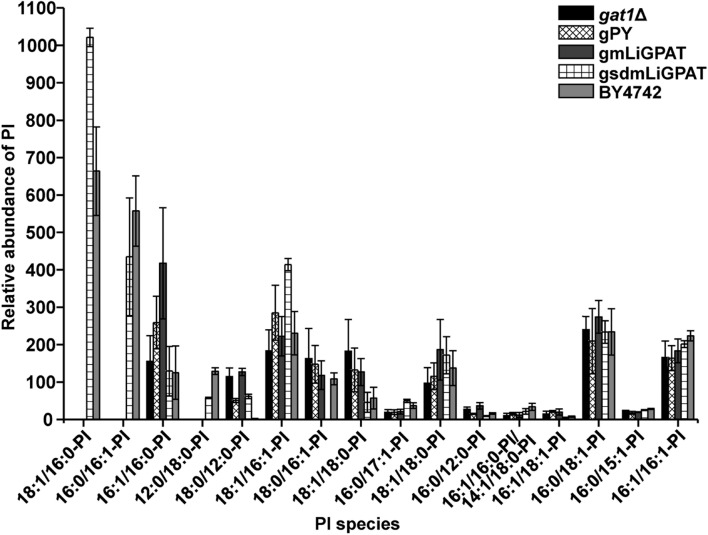
**Relative abundance of PI species in the top 35 VIP from *gat1*Δ, gPY, gmLiGPAT, gsdmLiGPAT, and BY4742**.

**FIGURE 4:**
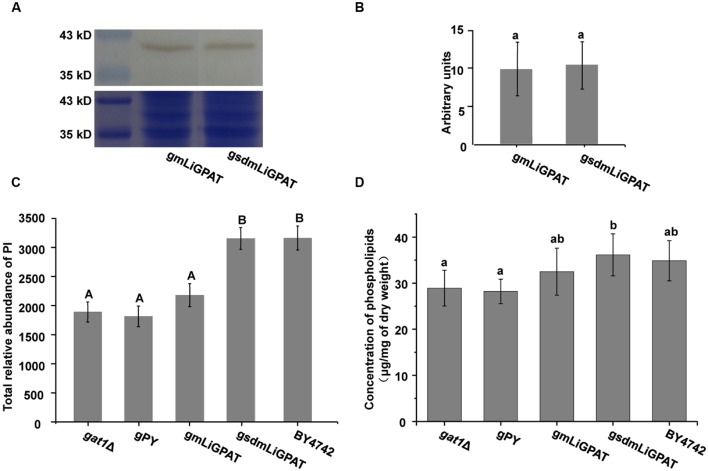
**The effect of site-directed mutagenesis of LiGPAT from Arg^195^ to His in yeast.** **(A)** Western blot analysis of gmLiGPAT and gsdmLiGPAT with the LiGPAT antibody. **(B)** The density of the blots of gmLiGPAT and gsdmLiGPAT were measured with ImageJ software and expressed in arbitrary optical density units. Values are the mean ± SD, *n* = 3. The average levels of gmLiGPAT and gsdmLiGPAT showed no difference (*P* > 0.05). **(C)** Comparison of the total relative abundance of all PI species among *gat1*Δ, gPY, gmLiGPAT, gsdmLiGPAT, and BY4742. Values with the same letter showed no significant difference (*P* > 0.05); the others showed significant differences (*P* < 0.01). **(D)** Comparison of the concentration of phospholipids among *gat1*Δ, gPY, gmLiGPAT, gsdmLiGPAT, and BY4742. Values with the same letter showed no significant difference (*P* > 0.05); the others showed significant differences (*P* < 0.05).

To understand which lipid mainly contributed to the separation of the wild-type group from the deficient group, 35 potential lipid biomarkers (VIP ranged from 38.34 to 2.59 with an average of 7.80) (**Table 1**) were selected according to both VIP values and the corresponding 95% confidence interval based on a jack-knife procedure (Eriksson et al., 2006). A total of 29 of these selected biomarkers were subsequently identified to be (PI, lyso-PI, PG, PS, PC, and PE (**Table 1**). Among these metabolites, PI accounted for 55.17% (16–29) and possessed relatively high VIP (ranging from 2.83 to 38.34 with an average of 9.613) (**Table 1**), indicating that this phospholipid was the main contributor to the separation. The relative abundance of most PI species from the wild-type group was higher than from the deficient group (**Figure 3**). This result was roughly consistent with the previous report (Redón et al., 2011) that the main increase of PI was observed when the yeast strain was cultured under low temperature. Therefore, the total relative abundance of PI species in the wild-type was compared with that in the deficient group. All of the PI species, 21 in total, were subsequently identified (**Table 2**), and the relative abundance was compared. The results showed that the total relative abundance of PI from gmLiGPAT was higher, although not significantly higher (*P* > 0.05), than from *gat1*Δ and gPY, whereas the total relative abundance of PI from gsdmLiGPAT was significantly higher (*P* < 0.01) than from gmLiGPAT, *gat1*Δ, and gPY, but there was no significant difference (*P* > 0.05) from BY4742 (**Figure 4C**). It was thus predicted that the site-directed mutagenesis of LiGPAT Arg195His might enhance the catalytic activity of this protein and result in an increase in the phospholipid level in yeast. The subsequent measurement of phospholipid content showed that the phospholipid level from gsdmLiGPAT was higher than from gmLiGPAT or BY4742 (**Figure 4D**), thus supporting the prediction.

**Table 1:** Identification of the top 35 metabolites contributing to differences between the wild-type group (gmLiGPAT, gsdmLiGPAT, and BY4742) and the deficient group (*gat1*Δ and gPY).

No	RT	m/z	VIP	Identification
1	11.11	835.5368	38.3386	18:1/16:0-PI
2	10.09	807.5041	27.1032	16:0/16:1-PI
3	6.82	339.2300	20.1118	Unknown
4	6.82	163.1099	16.6411	Unknown
5	10.30	807.5040	14.8547	16:1/16:0-PI
6	10.98	719.4896	11.6084	16:0/16:1-PG
7	9.97	781.4892	11.0091	12:0/18:0-PI
8	10.16	781.4890	8.2496	18:0/12:0-PI
9	10.72	833.5218	8.22112	18:1/16:1-PI
10	3.96	299.2570	8.06764	16:1/16:1-PE
11	11.66	835.5358	8.06364	18:0/16:1-PI
12	13.44	863.5690	6.98515	18:1/18:0-PI
13	11.04	821.5210	6.50701	16:0/17:1-PI
14	11.23	686.4771	5.83435	16:1/16:1-PE
15	10.98	745.5063	5.28594	18:1/16:1-PG
16	9.97	717.4710	5.25232	16:1/16:1-PG
17	10.16	717.4707	5.1447	16:1/16:1-PG
18	13.96	760.5160	5.11763	18:1/16:0-PS
19	13.63	760.5144	5.00298	16:0/18:1-PS
20	12.45	688.4934	4.12377	16:0/16:1-PE
21	2.80	271.2242	4.03169	Unknown
22	13.27	863.5690	4.02396	18:1/18:0-PI
23	9.33	753.4565	3.96868	16:0/12:0-PI
24	11.02	807.5054	3.78887	16:1/16:0-PI or
				14:1/18:0-PI
25	4.60	353.2115	3.77584	Unknown
26	10.84	774.5312	3.75723	18:1/17:0-PS
27	19.04	1114.7429	3.68253	Unknown
28	11.36	833.5210	3.52542	16:1/18:1-PI
29	12.85	714.5086	3.37159	16:1/18:1-PE
30	12.06	835.5378	3.33699	16:0/18:1-PI
31	21.81	710.6666	3.1069	Unknown
32	10.00	793.4881	3.02204	16:0/15:1-PI
33	9.63	805.4902	2.82583	16:1/16:1-PI
34	10.14	722.4961	2.74706	12:0/16:0-PC
35	3.35	478.2912	2.59477	18:1-lysoPE


**Table 2:** List of identified PI species in the ESI^-^ model.

No	RT	m/z	Identification
1	6.72	723.4066	16:1/10:0-PI
2	7.68	725.4233	16:0/10:0-PI
3	9.32	753.4564	16:0/12:0-PI
4	9.00	753.4598	12:0/16:0-PI
5	9.57	779.4742	14:1/16:0-PI or
			14:0-16:1-PI or
			12:0/18:1-PI
6	10.15	781.4889	18:0/12:0-PI
7	9.97	781.4892	12:0/18:0-PI
8	10.00	793.4881	16:0/15:1-PI
9	9.61	805.4915	16:1/16:1-PI
10	10.30	807.504	16:1/16:0-PI
	10.21	807.5042	
	10.58	807.5052	
	11.01	807.5056	
11	10.09	807.5059	16:0/16:1-PI
12	10.14	819.5068	16:1/17:1-PI
13	11.03	821.5215	16:0/17:1-PI
14	11.35	833.5210	16:1/18:1-PI
15	10.70	833.5219	18:1/16:1-PI
16	11.66	835.5358	18:0/16:1-PI
17	11.11	835.5367	18:1/16:0-PI
18	12.06	835.5378	16:0/18:1-PI
19	12.47	861.5568	18:1/18:1-PI
20	13.43	863.5687	18:1/18:0-PI
	13.27	863.5690	
21	10.01	875.4960	16:0/21:2-PI


## Discussion

### Plastid-Localized GPAT from *L*. *incisa*

Glycerol-3-phosphate acyltransferases targeting the chloroplast, cytoplasm, and mitochondrion have been recognized in plants. The chloroplastidial GPAT localized in the stroma is a soluble protein, and it can utilize acyl-(acyl-carrier protein) as the acyl donor (Joyard and Douce, 1977). In contrast, the cytoplasmic form targeted to the ER is hydrophobic, and it is able to utilize acyl-CoA as the acyl donor (Frentzen et al., 1990). Genes of both chloroplastidial and ER-bound GPAT from several higher plants have been cloned, a total of 10 from *Arabidopsis* (Zheng et al., 2003; Xu et al., 2006; Gidda et al., 2009; Cagliari et al., 2010; Chen et al., 2010; Yang et al., 2012), 9 from *Ricinus communis* (Cagliari et al., 2010), and at least 2 from *Glycine max* (Eskandari et al., 2013), for instance. Examination of the algal genomes indicated that the microalgae *Chlamydomonas reinhardtii*, *Ostreococcus tauri*, *Cyanidioschyzon merolae* strain 10D, *Phaeodactylum tricornutum* CCAP 1055/1, and *Thalassiosira pseudonana* CCMP1335 were missing the recognizable extraplastidial GPAT homologs (Lykidis and Ivanova, 2008). It was suggested that the GPATs in these microalgae might have dual localization in both chloroplast and ER (Lykidis and Ivanova, 2008).

The present study provides the convincible bioinformatics evidence that one GPAT from the green microalga *L*. *incisa* is localized to chloroplasts (**Figures 1A,C**). In the latest NCBI database, there were deposited putative green microalgal GPATs, which were similar to the ER-bound GPAT9 from *Arabidopsis*. Therefore, it was inferred that the GPATs from *L*. *incisa* and the above-mentioned microalgae might be only localized to chloroplasts. Obviously, this idea would be more convincing and significant with analysis of accurate subcellular localization of the chloroplastidial GPATs and function of the cytoplasmic ones from these green microalgae.

### Site-Directed Mutagenesis of LiGPAT Resulted in an Increase of the Phospholipid Level in Yeast

The phospholipid level of the yeast transformed with the site-directed mutated *LiGPAT* (Arg195His) was higher than that of yeast transformed with the original *LiGPAT* (**Figure 4D**), indicating that the catalytic ability of LiGPAT was improved by site-directed mutagenesis. To explore whether this improvement resulted from an increased level of protein expression or an increased enzymatic activity, semi-quantitative analysis of LiGPAT introduced into yeast was performed using western blots with purified LiGPAT polyclonal antibody. The reliability of this antibody was supported by western blot analysis of the total proteins extracted from *L*. *incisa* and transformed *E*. *coli* pmLiG/BL (**Supplementary Figure S2**). Comparison of the band intensity on the blots indicated that the expression levels of mLiGPAT and sdmLiGPAT were not significantly difference (*P* > 0.05) (**Figures 4A,B**), suggesting that the site-directed mutagenesis from Arg^195^ to His had no effect on protein expression level but could enhance the enzymatic activity of LiGPAT. This prompted us to investigate the relationship between protein structure and enzymatic activity of LiGPAT because the mutated residue Arg is situated in the G-3-P binding pocket (**Figure 1A**).

**FIGURE 5:**
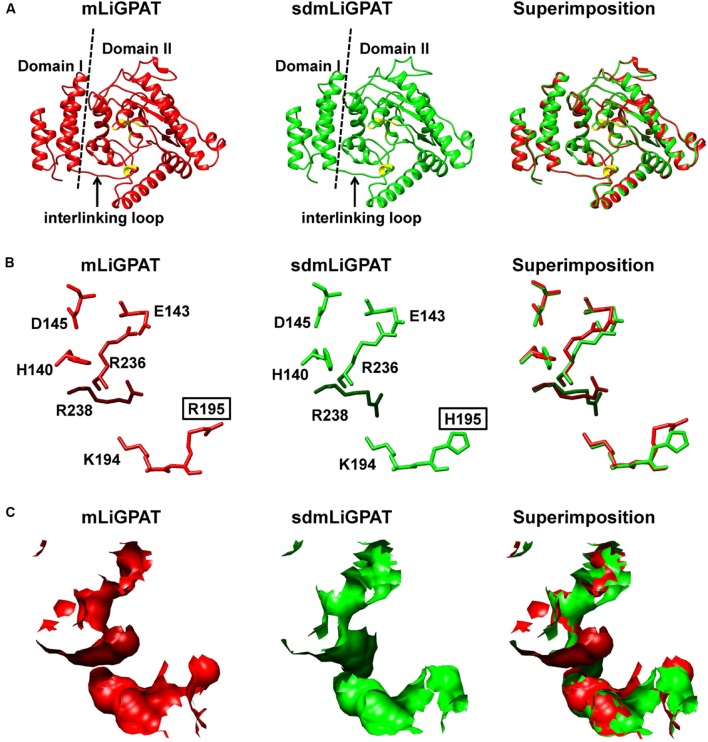
**Superimposition of 3D structure models of the LiGPAT mature protein (red) and its mutant (Arg195His) (green).** **(A)** Schematic ribbon representation showing the arrangements of α-helices, β-sheets, and loops. Domain I (left) and Domain II (right) are separated by the dashed line. The “interlinking loop” region is indicated by arrow. Residues composing the positively charged G-3-P binding pocket are in yellow. Atom **(B)** and surface **(C)** representation displaying the superimposition of the positively charged G-3-P binding pocket.

The crystal structure of squash chloroplastidial GPAT protein (PDB entry 1K30) was the only structure solved with high-resolution that elucidated the structure-function relationship of GPAT (Heath and Rock, 1998; Turnbull et al., 2001; Tamada et al., 2004). Accordingly, 3D models of mLiGPAT and sdmLiGPAT (**Figure 5**) were developed by using the I-TASSER server, which was an integrated platform for automated protein structure and function prediction based on the sequence-to-structure-to-function paradigm (Roy et al., 2010). The secondary structural elements of the mLiGPAT and sdmLiGPAT proteins were organized into two domains (**Figure 5A**), which were found well conserved in *Cucurbita moschata*, *Chlamydomonas reinhardtii*, *Arabidopsis thaliana*, and *Glycine max* (Turnbull et al., 2001; Misra and Panda, 2013). Domain I is the GPAT_N, and it forms a four-helix bundle (consisting of the 3_10_ helix linking residues 7–10 and helices α1–3) with a simple square, right-handed up–down-up–down topology (**Figures 1A** and **5A**). A loop region called the “interlinking loop” linked the small Domain I and the large Domain II. Domain II comprises the alternating α/β secondary structural elements (**Figure 5A**) and positively charged residues, which constitutes a positively charged pocket for binding the phosphate group of G-3-P. These residues were well conserved in most plants, except that the His residue at position 195 in mLiGPAT was substituted by Arg (**Figure 1A**). However, this replacement did not change the charge property and secondary structure of the pocket from mLiGPAT compared to that from its site-directed mutant sdmLiGPAT (**Figure 5A**). Structural superimposition of the binding sites for the phosphate group of G-3-P from mLiGPAT and sdmLiGPAT was illustrated by atom (**Figure 5B**) and surface (**Figure 5C**). The modeling indicated that the side-chain conformation of residues at positions 195 and 238 were different between these two proteins, suggesting a smaller accessible surface of the phosphate group binding pocket from mLiGPAT than from sdmLiGPAT. Therefore, changes of the side-chain conformation might be responsible for the difference in the enzymatic activity of mLiGPAT and sdmLiGPAT when they function in yeast.

In brief, acylation by GPAT is considered to be the rate-limiting step in the glycerolipid synthesis pathway and to regulate fatty acid flux through the pathway (Coleman and Lee, 2004; Wendel et al., 2009). In *Arabidopsis*, RNAi of the chloroplastidial *GPAT* in the *ats1-1* mutant background led to small leaves (Xu et al., 2006). Thus, it was inferred that the low growth rate of *L*. *incisa* (Ouyang et al., 2013b) might be partially associated with the relatively low enzymatic activity of LiGPAT. Recently, a modification of the GPAT-coding gene together with four other genes has been documented to improve the TAG content in *Chlorella minutissima* UTEX 2219 (Hsieh et al., 2012). Hence, genetic manipulation of the G-3-P binding sites of *GPAT* could be taken as a breakthrough to increase the growth rate and glycerolipid content of *L*. *incisa* and other microalgae.

## Author Contributions

Z-GZ and L-LO designed the study and wrote the paper. L-LO carried out the experiments and she and Z-GZ were involved in data analysis. HL assisted with heterologous expression of *LiGPAT* in yeast. X-JY and J-LX helped design the lipidomic experiments and interpret the data. Z-GZ gave the final approval of the version to be published. All authors have read and approved the final manuscript.

## Conflict of Interest Statement

The authors declare that the research was conducted in the absence of any commercial or financial relationships that could be construed as a potential conflict of interest.

## Footnotes

2 http://blast.ncbi.nlm.nih.gov/

3 http://www.ncbi.nlm.nih.gov/spidey/

4 http://www.cbs.dtu.dk/services/SignalP/

5 http://www.cbs.dtu.dk/services/TargetP/

6 http://www.cbs.dtu.dk/services/ChloroP/

7 http://www.predictprotein.org

8 http://zhanglab.ccmb.med.umich.edu/I-TASSER/

9 http://wishart.biology.ualberta.ca/SuperPose/

10 http://web.uni-frankfurt.de/fb15/mikro/euroscarf/

11 rsb.info.nih.gov/ij/

12 http://www.hmdb.ca

13 http://www.lipidmaps.org

14 http://metlin.scripps.edu

## Abbreviations

AA: arachidonic acid
CDD: Conserved Domain Database
CIJF_JK_: jack-knifed confidence interval
cTP: chloroplast transit peptide
CV-ANOVA: cross-validated analysis of variance
DAB: diaminobenzidine
ER: endoplasmic reticulum
G-3-P: Glycerol-3-phosphate
GPAT: Glycerol-3-phosphate acyltransferase
LPAAT: lysophosphatidic acid acyltransferase
m/z: mass-to-charge ratio
NC: nitrocellulose
OPLS-DA: orthogonal projection to latent structures with discriminant analysis
PC: phosphatidylcholine
PCA: principal components analysis
PE: phosphatidylethanolamine
PG: phosphatidylglycerol
PI: phosphatidylinositol
PLS-DA: projection to latent structures with discriminant analysis
PS: phosphatidylserine
RT: retention time
SDS-PAGE: SDS polyacrylamide gel electrophoresis
TAG: triacylglycerol
TAP: Tris acetate phosphate
UPLC-ESI-Q-TOF-MS: ultra performance liquid chromatography-electron spraying ionization-qadrupole-time-of-flight-mass spectrometry
VIP: variable importance in the projection
