# Supplementary material for: Site-Directed Mutagenesis from Arg195 to His of a Microalgal Putatively Chloroplastidial Glycerol-3-Phosphate Acyltransferase Causes an Increase in Phospholipid Levels in Yeast
Source: Front Plant Sci. 2016 Mar 10;7:286. doi: 10.3389/fpls.2016.00286 (PMC4785142; doi:10.3389/fpls.2016.00286)
Supplement: Supplementary file 1 [file Table_1.DOC]

**Supplementary Table 1 Primers applied for LiGPAT gene cDNA cloning, DNA cloning, southern blot, and heterologous expression.**

| **Primes** | **Nucleotide sequences (5′-3′)** |
| --- | --- |
| **cDNA cloning** |  |
| G1 | TACAMSTTCCCCAGCTTCCAC |
| G2 | CAGGTYTYTGCCCATGCTGAAG |
| NGSP5-1* | GCTGCTCAATCTCATCAAACCGTGCT |
| GSP5-2 | GCCACATAGATGACGTCAGTCGCCA |
| GSP5-4 | CCTTTGGAGTGGCTGCCTATGTTGTG |
| NGSP3-1* | TACACGTTCCCCAGCTTCCACCAC |
| GSP3-2 | CCACCGCATCCTGGAGCCCTACAACT |
| **DNA cloning** |  |
| P1 | F: ATTAAGCAGTGGTATCAACG |
|  | R: CGCCGTACAGATTTGACCA |
| P2 | F: ATTGGCAGCATAAGGTTGCAC |
|  | R: ACAGCTCGCAGTAGGTACG |
| P3 | F: AACGTGGCTCATTACGGCTGCAT |
|  | R: TTGGAGTGGCTGCCTATGTTGTG |
| P4 | F: ATCTGACACTTGCAACCGTACAGC  R: TTGTCCAACTCGTCATCACATGCT |
| **Southern blot** | |
| SOB | F: AACCACCAGACGGAGGCAGAC |
|  | R: TCTTGGAGTGCACGCAGAAGAG |
| **Heterologous expression** | |
| EcBamF | GGATCCATGGCACAGGTCGTCCGCCAGAAG |
| ScBamF | GGATCCAACATGGCACAGGTCGTCCGCCAGAAG |
| MuScF | TGCACTCCAAGAAGC ACCTGGACGACATCC |
| MuScR | TCCAGGTGCTTCTTGGAGTGCACGCAGAA |
| XhoR | CTCGAGTTACTGGGCCCATGGCTGCTGGTA |
| pYF | AACCCCGGATCGGACTACTAG |
| pYR | CTTTTCGGTTAGAGCGGATG |

* presents the nest gene specific primers for RACE reaction.

The digestion sites are underlined.
