# Supplementary material for: Site-Directed Mutagenesis from Arg195 to His of a Microalgal Putatively Chloroplastidial Glycerol-3-Phosphate Acyltransferase Causes an Increase in Phospholipid Levels in Yeast
Source: Front Plant Sci. 2016 Mar 10;7:286. doi: 10.3389/fpls.2016.00286 (PMC4785142; doi:10.3389/fpls.2016.00286)
Supplement: Supplementary file 2 [file Image_1.PDF]

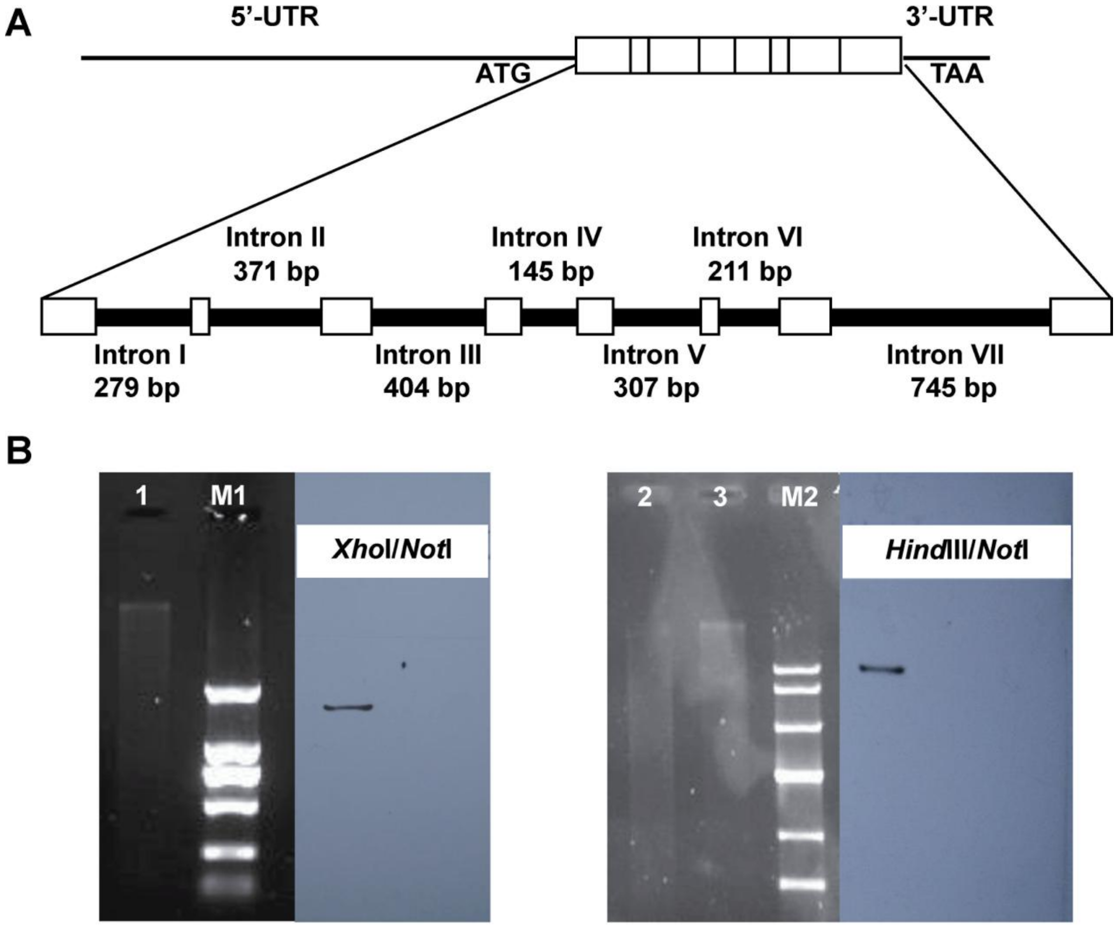

Supplementary Figure 1 Gene structure and the copy number of GPAT gene from *Lobosphaera incisa*. (A) The box represents extrons. A total of 7 introns with length of 279 bp, 371 bp, 404 bp, 145 bp, 307 bp, 211 bp, and 745 bp, respectively, are presented as the filled box. Bold lines represent 5′-UTR and 3′-UTR. **(B)** Agarose gel electrophoretogram of *L. incisa* genomic DNA digested by *Xho*I/*Not*I (Lane 1) and *Hind*III/*Not*I (Lane 2) and the corresponding southern blot results are shown. M1 and M2 represent D2000 DNA marker (Tiangen) and DNA marker IV (Tiangen), respectively.
