# Supplementary material for: Site-Directed Mutagenesis from Arg195 to His of a Microalgal Putatively Chloroplastidial Glycerol-3-Phosphate Acyltransferase Causes an Increase in Phospholipid Levels in Yeast
Source: Front Plant Sci. 2016 Mar 10;7:286. doi: 10.3389/fpls.2016.00286 (PMC4785142; doi:10.3389/fpls.2016.00286)
Supplement: Supplementary file 3 [file Image_2.PDF]

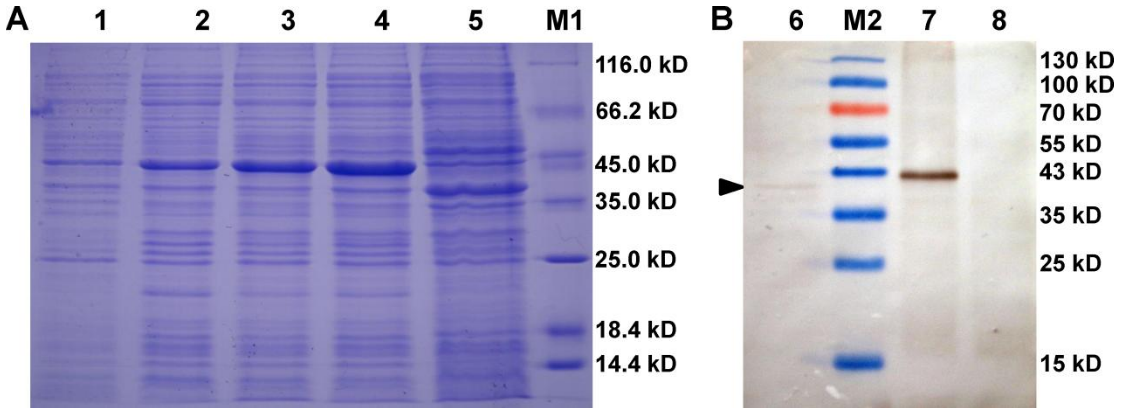

Supplementary Figure 2 Heterologous expression of *LiGPAT* in *Escherichia coli* and purified LiGPAT polyclonal antibody validation by western blot analysis. (A) Electrophoresis of recombinant LiGPAT in *E. coli* induced by IPTG for different times. M1: Unstained protein molecular weight marker (Thermo-Fisher). M2: Prestained protein molecular weight marker (Thermo-Fisher). Lane 1–4: Expression of *LiGPAT* induced by IPTG for 0 h, 2 h, 4 h, and 6 h. Lane 5: Expression of pET 28a induced by IPTG for 6 h. HPLC-MS analysis showed that the recombinant protein matched the LiGPAT protein as a representative fragment at m/z 1,292.6942 was identical to the partial amino acid sequence (LATDVIYVAGDR) of LiGPAT. **(B)** Western blot analysis of the total proteins extracted from *L. incisa* (Lane 6), transformed *E. coli* pmLiG/BL (Lane 7), and plasmid-only transformed *E. coli* pET/BL (Lane 8). The filled arrow indicates the single band detected in *L. incisa* (Lane 6).
